# Exploring Primary Care Patients’ Perspectives on Artificial Intelligence: Systematic Literature Review and Qualitative Meta-Synthesis

**DOI:** 10.2196/72211

**Published:** 2025-11-19

**Authors:** Alisa Mundzic, Robin Bogdanffy, David Sundemo, Pär-Daniel Sundvall, Jonathan Widén, Peter Nymberg, Carl Wikberg, Anna Moberg, Ronny Gunnarsson, Artin Entezarjou

**Affiliations:** 1 General Practice/Family Medicine, School of Public Health and Community Medicine, Institute of Medicine, Sahlgrenska Academy, University of Gothenburg, Guldhedsgatan 5A, Gothenburg, 413 20, Sweden, 46 768969024; 2 Center for Digital Health, Sahlgrenska University Hospital, Sahlgrenska University Hospital, Gothenburg, Region Västra Götaland, Sweden; 3 Lerum Primary Health Care Center, Närhälsan, Lerum, Sweden; 4 Research, Education, Development & Innovation, Primary Health Care, Region Vastra Gotaland, Gothenburg, Sweden; 5 Center for Primary Health Care Research, Department of Clinical Sciences, Lund University, Malmö, Sweden; 6 Health Care Centre Laröd, University Clinic Primary Care, Skåne University Hospital, Region Skåne, Lund, Sweden; 7 Department of Health, Medicine and Caring Sciences, Linkoping University, Linköping, Sweden; 8 Primary Health Care Center Kärnan, Region Östergötland, Linköping, Sweden; 9 Centre for Antibiotic Resistance Research (CARe), University of Gothenburg, Gothenburg, Sweden

**Keywords:** artificial intelligence, large language models, natural language processing, generative AI, machine learning, primary health care, patient perspectives, systematic review, qualitative meta-synthesis

## Abstract

**Background:**

The introduction of artificial intelligence (AI) in health care holds great promise, offering the potential to alleviate physicians’ workloads and allocate more time for patient interactions. After the emergence of large language models (LLMs), interest in AI has surged in the health care sector, including within primary care. However, patients have expressed concerns about the ethical implications and use of AI in primary care. Understanding patients’ perspectives on using AI in primary care is crucial for its effective integration. Despite this, few studies have addressed patients’ perspectives on using AI in primary care.

**Objective:**

This study aimed to synthesize qualitative research on primary care patients’ perspectives regarding the use of AI, including LLMs, in primary care.

**Methods:**

A qualitative systematic review, using thematic analysis, was performed in accordance with PRISMA (Preferred Reporting Items for Systematic Reviews and Meta-Analyses) guidelines. Databases, including PubMed, Scopus, Web of Science, CINAHL, and PsycINFO, were searched from inception to February 5, 2024. Eligible studies (1) used a qualitative interview research design, (2) explored primary care patients’ perspectives on the use of AI in primary care, (3) were written in English, and (4) were published in peer-reviewed scientific journals. Quantitative studies, gray literature, surveys, and studies lacking depth in qualitative analysis were excluded. The Critical Appraisal Skills Program (CASP) checklist was used for quality assessment.

**Results:**

Of 1004 studies screened, 6 were included, comprising 170 patients aged 13-91 years from 3 countries. Three themes emerged: “The Relationship with and Actions of AI Systems,” “Implementing AI responsibly,” and “Training Physicians and Artificial Minds.” Patients acknowledged AI’s potential benefits but advocated for clinician oversight, safety frameworks, and the preservation of patient autonomy.

**Conclusions:**

This systematic review provides an understanding of patients’ perspectives on AI in primary care. We identified heterogeneity in AI definitions across studies. Further research is needed on patients’ perspectives across different countries. Notably, our synthesis revealed a significant research gap, as none of the included studies particularly explored patients’ perspectives on LLMs, highlighting an important area for future research.

## Introduction

### Background

The health care sector is facing challenges, with high administrative workloads and patients growing older and becoming more vulnerable. Primary care, often the first point of contact with health care, is especially vulnerable to these challenges [1]. Introducing artificial intelligence (AI) in primary care is suggested to free up time for patient contact and prevent physician burnout through the automation of administrative tasks [2,3].

Today, AI-assisted tools are widely used in various clinical settings and can successfully help predict mortality risks [4], improve therapy, and assist diagnosis [5]. Algorithms that are being used are already outperforming clinicians in spotting malignant tumors [6-9]. AI solutions are predominantly implemented in referral care settings, with limited use in primary care [10]. The most common AI applications in primary care include diagnostic decision support, treatment decision support, and data extraction [10].

Introducing AI in health care, however, is not without risks. Discrimination through biased training data, uncertainty regarding the division of moral and legal responsibilities, and questions of integrity and privacy are all concerns that may limit the implementation of AI in health care [10,11]. In addition, implementing AI in health care brings ethical obligations, as it both directly and indirectly impacts patient care [12].

Patient engagement is increasingly being recognized as vital to health care delivery, improving outcomes, safety, and reducing hospital admissions and costs [13]. Impactful organizations, including the World Health Organization (WHO) and the Organization for Economic Co-operation and Development (OECD), have identified patients as one of the main stakeholders in the development and assessment of medical AI applications [14,15]. For AI implementation to be successful, it is essential to consider the needs and perspectives of the end users. While studies have been conducted on clinicians’ perspectives of AI in primary care [16-26], few studies exist on primary care patients’ perspectives on AI [27-30].

The emergence of large language models (LLMs) has significantly advanced the capabilities of AI systems, particularly in their ability to interpret and generate human language [31]. These models, such as ChatGPT (OpenAI), are built on deep learning (DL) and natural language processing (NLP) [31], and their conversational interfaces introduce new challenges and opportunities in health care [32,33]. In primary care, where the patient-doctor consultation is central, the integration of LLMs may influence communication, decision-making, and the overall care experience [34]. As LLMs become increasingly integrated into health care systems, it is important to consider how patients perceive and interact with these technologies. Understanding patients’ perspectives on AI, including emerging forms, such as LLMs, is essential for responsible and effective implementation.

To our knowledge, this is the first qualitative meta-synthesis aiming to target primary care patient perspectives on AI and LLMs in primary care.

### Aim

The primary objective of this qualitative meta-synthesis is to synthesize the current qualitative research on primary care patients’ perspectives on the use of all types of AI, including the use of LLMs, in primary care.

## Methods

### AI Definitions

AI encompasses a diverse array of technologies rather than being just a single innovation [6]. The challenge in defining AI technology stems from the lack of a clear, consensus-driven definition. The types of technology associated with the term AI appear to shift over time, creating difficulties in capturing patients’ perspectives [35]. One influential definition has been presented by the OECD: “An AI system is a machine-based system that, for explicit or implicit objectives, infers, from the input it receives, how to generate outputs, such as predictions, content, recommendations, or decisions that can influence physical or virtual environments. Different AI systems vary in their levels of autonomy and adaptiveness after deployment” [36].

**Figure 1.**
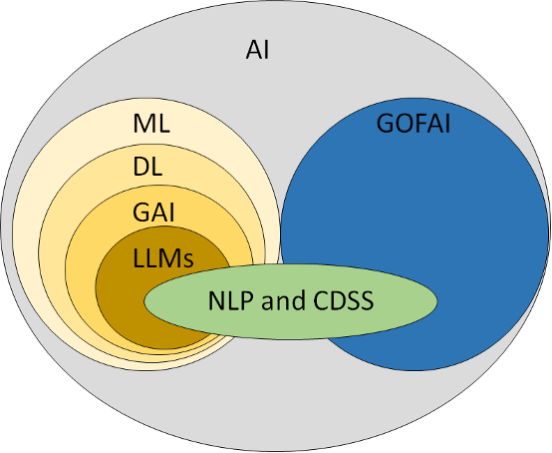
Various AI systems with overlapping characteristics. NLP and CDSS applications may be based on either ML or good old-fashioned AI architectures. AI: artificial intelligence; CDSS: clinical decision support systems; DL: deep learning; GAI: generative artificial intelligence; GOFAI: good old-fashioned AI; LLM: large language models; ML: machine learning; NLP: natural language processing.

The increasing integration of AI in health care has introduced various systems and models with overlapping and complex characteristics, as presented in Figure 1. To give context to the different types of AI technologies that may be relevant in primary care, we include short descriptions of key concepts, such as machine learning (ML), DL, NLP, LLMs, and clinical decision support systems (CDSS). These definitions are included to help the reader understand how patients might relate to different AI systems and to reflect how the field of AI is developing in health care. ML uses algorithms to learn patterns, and by training on large datasets, it can make predictions based on the training data. DL, a complex form of ML, can predict outcomes based on many different variables. NLP, based on ML (mostly DL), recognizes and analyzes texts and speech [6]. NLP allows computers to interpret and generate human language [37]. LLMs involve the use of NLP and DL to interpret and generate human language based on the input they receive [31]. LLMs may serve as foundation models for developing more sophisticated AI applications, such as ChatGPT (OpenAI). Further technical details and definitions of AI are provided in Multimedia Appendix 1 [6,37-42].

### Study Design

This review was prospectively registered with the International Prospective Register for Systematic Reviews (PROSPERO; CRD42024505209) [43] and is reported in accordance with PRISMA (Preferred Reporting Items for Systematic Reviews and Meta‐Analyses) statement [44]. A completed PRISMA checklist is provided in Checklist 1.

This review used a systematic review methodology for qualitative studies, where the authors conducted a secondary qualitative analysis of the published quotes and supplementary information from the reviewed studies, allowing for a deeper exploration of underlying patterns and themes.

### Search Strategy

Searches for peer-reviewed literature were conducted in PubMed and Scopus, with the assistance of a research librarian and research colleagues. Broader terms for “primary health care” and “artificial intelligence” ensured an inclusive search string. In PubMed, the search incorporated MeSH (Medical Subject Headings) terms and Boolean operators. The finalized search string was registered in PROSPERO before conducting the final search on February 5, 2024. After feedback from reviewers, an extended search was conducted in Web of Science, CINAHL, and PsycINFO on July 10, 2025. To ensure a comprehensive review, the reference lists of eligible studies were also screened to identify any studies that may have been missed in our initial searches. The search strings are listed in Multimedia Appendix 2.

### Study Selection

The initial search results were imported into Rayyan (Rayyan Systems Inc) software [45] for duplicate removal, with all duplicates being manually verified and excluded from the review. Two authors, A Mundzic and RB, evaluated the studies for inclusion, independently screened the studies based on titles and abstracts, and performed a full-text evaluation. Any discrepancies during the inclusion process, whether in the title and abstract screening or full-text evaluations, were resolved through discussion. In this review, 6 studies [1,2,12,17,46,47] were included, with 4 studies [1,12,46,47] exclusively focusing on patients’ perspectives and 2 studies [2,17], including both patients’ and clinicians’ perspectives. Clinicians’ opinions were excluded during coding.

### Inclusion and Exclusion Criteria

Inclusion criteria were defined using the SPIDER (sample, phenomenon of interest, design, evaluation, research type) framework [48]. Studies were eligible if they (1) used a qualitative interview-based design, (2) were written in English, (3) were published in peer-reviewed scientific journals, (4) were conducted in a primary health care setting, and (5) focused on patients’ perspectives on the use of AI in primary care. For this review, “conducted in a primary health care setting,” was defined as studies in which participants were recruited through primary care, had documented contact with primary care, or where the study context explicitly involved primary care (eg, general practice and family medicine). Studies were excluded if they were quantitative, survey-based, gray literature, or lacked sufficient depth in qualitative analysis. Consequently, studies lacking patient quotations (eg, survey-only or author-summarized reports) were excluded for not providing sufficient insight into participants’ experiences.

### Data Extraction and Quality Assessment

A Mundzic extracted all data from the eligible studies within the results and supplementary information that contained opinions and direct quotations from patients. A summary of the characteristics of the included studies was compiled by A Mundzic, and the second author, RB, reviewed the table for accuracy. The following data were extracted: number of participants, interview dates, explanation of AI, type of AI in the study, and emerging themes. In case of missing information relating to the study objectives, the corresponding authors of the respective publications were contacted.

The methodological quality of the chosen studies was assessed using the Critical Appraisal Skills Program Qualitative Research Checklist (CASP) [49]. The authors A Mundzic and RB, independently conducted the quality appraisal, after which they compared their findings and sorted out any discrepancies by discussion. In cases of disagreement, a discussion was held, and if consensus could not be reached, a third researcher, AE, was consulted to finalize the rating as “YES,” “NO” or “Can’t tell.” In cases where information was missing, the authors answered, “Can’t tell.” The CASP checklist is present as Checklist 2.

### Data Analysis and Synthesis

Coding was performed in 2 steps. First, the 4 studies [1,12,46,47] focusing exclusively on patients’ perspectives were coded. The remaining 2 studies [2,17], which included both patients’ perspectives and clinicians’ opinions, were coded separately. The emerging themes from these mixed studies were then compared to those from the studies focusing solely on patients to determine whether the presence of clinicians influenced the themes. No differences were found, and all the patients’ perspectives are presented in this report.

The coding was conducted by A Mundzic, with RB double-checking the codes. Any discrepancies were resolved through discussion before proceeding. In cases of disagreement, a third researcher, AE, was consulted. Both authors independently grouped the codes and discussed emerging themes before finalizing them (see Multimedia Appendix 3 for the full codebook). NVivo 14 software (Lumivero) was used to generate initial codes, search for themes, and identify final themes. Thematic analysis was performed using an inductive approach, following Braun and Clarke’s [50] 6-step method to identify latent themes. Inductive thematic analysis with a latent approach offers a flexible and in-depth data analysis [51]. Since patients’ perspectives on AI in primary care are a relatively unexplored area of research [46], this method is suitable to answer the research question.

### Ethical Considerations

Ethical approval is not required for this systematic review, as it is based solely on previously published studies. All included studies received ethical approval, except for the study conducted in Denmark, where ethical approval was deemed unnecessary according to Danish national regulations, as it involved self-reported data.

## Results

### Search Results and Selection

**Figure 2.**
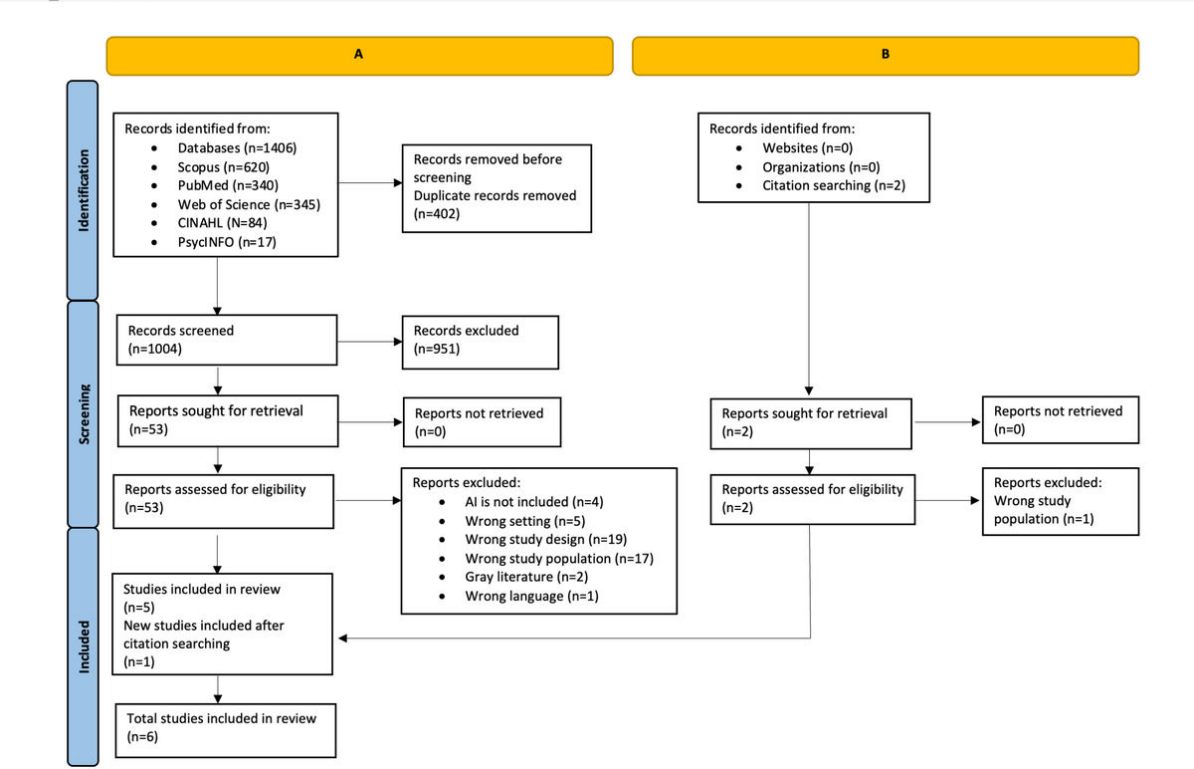
PRISMA (Preferred Reporting Items for Systematic Reviews and Meta-Analyses) flowchart of study selection. AI: artificial intelligence. (A) Identification of studies via databases and registers. (B) Identification of studies via other methods.

A total of 1406 records were retrieved from the electronic databases. After removing duplicates, 1004 studies remained for abstract and title screening. Following title and abstract screening, 53 full-text studies were screened, with 1 additional study identified from reference screening of the included studies. In this review, 6 studies [1,2,12,17,46,47] were included. Among them, 4 studies [1,12,46,47] exclusively focused on the patients’ perspectives, and 2 studies [2,17] included the perspectives of patients, where clinicians’ opinions were present. The process of the study selection is shown in Figure 2.

### Study Characteristics

The characteristics of the 6 included studies [1,2,12,17,46,47] are visualized in Table 1, and additional information is presented in Multimedia Appendix 4.

Four [12,17,46,47] of the included studies were conducted in the United States, and one each was conducted in Denmark [1] and Canada [2]. Two studies [12,46] were conducted by the same authors and appear to include the same participants but with different research questions. The ages of participants ranged from 13 to 91 years. Interviews were held between October 2019 and January 2022. The publication dates of the included studies ranged from September 21, 2021 to January 6, 2024. A total of 170 unique participants were included, excluding those who overlapped across studies.

The 6 included studies [1,2,12,17,46,47] had different approaches to how AI was defined to the participants. In 2 of the studies [12,46], participants were given a brief definition of AI and case studies focused on using AI for image analysis, optimizing preventative health, in-patient monitoring, diagnostic support, and engagement with patients during primary care appointments. In 1 study [1], participants had little knowledge about AI. Different scenarios were used to explore patients’ opinions. In another study [47], questions were asked to explore participants’ perspectives on teleophthalmology, AI-based image interpretation, and virtual care. In the study [47], teleophthalmology was implemented as a part of a federally qualified health center’s primary care services, and the technology was used in routine diabetes care. The intervention was regarded as an extended service of general practice and therefore fell within the scope of the review. In the study by Davis et al [17], AI was described as integrating data from electronic health records and smartphones to predict suicide risk. In the last study [2], AI was described to participants as a technology that automates tasks typically requiring human intelligence, such as processing information, reasoning, learning, planning actions, and communicating in natural language, and presented as a general-purpose prediction technology that estimates missing information from available data.

**Table 1.** Sociodemographic and study characteristics.

Study	Age (years)	Sex n (%)	Participants, n	Type of AI^a^	Themes
A Framework for Examining Patient Attitudes Regarding Applications of Artificial Intelligence in Healthcare [12]	18-91	Female: 43 (49) Male: 44 (51)	87	Machine learning, including deep learning	Experiences Beliefs about health care and beliefs about technology Attitude toward AI in health care
Patient Perspectives on Data Sharing Regarding Implementing and Using Artificial Intelligence in General Practice: A Qualitative Study [1]	25-74	Female: 6 (60) Male: 4 (40)	10	Unspecified	Patient-General Practitioner relationship Willingness for data sharing Worries about data sharing AI’s possibilities in general practice Worries about the use of artificial intelligence in general practice
Perspectives of Latinx Patients with Diabetes on Teleophthalmology, Artificial Intelligence-Based Image Interpretation, and Virtual Care: A Qualitative Study [47]	33-79	Female: 12 (60) Male: 8 (40)	20	Unspecified (AI-based image interpretation)	Teleophthalmology is better at detecting eye disease than an in-person eye exam Teleophthalmology allows the patient to see their screening results with the eye photo Teleophthalmology doesn’t require dilating eye drops Teleophthalmology is more affordable, convenient, and time-efficient Teleophthalmology complements the in-person physical exam by the PCP ^b^ Distrust of technology Preference for following a doctor’s recommendation regarding the method of diabetic eye screening Preference for a human physician to review eye photos versus AI Preference for a human physician to use AI for decision support but make the final diagnosis Ambivalence toward AI-based eye screening Virtual care removes barriers to care due to being more convenient and time-efficient Inability to adequately assess body language during a virtual care encounter Virtual care isn’t adequate when a patient needs a physical exam Virtual care should be used only when it is not possible to obtain in-person care Discomfort with virtual care due to lack of experience with using it Importance of in-person care for establishing an emotional connection with the doctor
Patient Apprehensions About the Use of Artificial Intelligence in Healthcare [46]	18-91	Female: 43 (49) Male: 44 (51)	87	Natural language processing and machine learning, including deep learning	Participants were excited about health care AI but wanted assurances about safety Patients expect their clinicians to ensure AI safety Preservation of patient choice and autonomy Concerns about health care costs and insurance coverage Ensuring data integrity Risks of technology-dependent systems
Adolescent, Parent, and Provider Perceptions of a Predictive Algorithm to Identify Adolescent Suicide Risk in Primary Care [17]	13-16	Female: 5 (56) Male: 4 (44)	31 in total; 9 adolescents 12 parents 10 providers	Machine learning	Privacy Clarification or Confusion Patient, family, and provider characteristics and experiences Clinical utility Communication Data and evidence Suggestions Inner setting Outer setting Acceptability
Priorities for Artificial Intelligence Applications in Primary Care: A Canadian Deliberative Dialog with Patients, Providers, and Health System Leaders [2]	23-73	Female: 12 (55) (including one trans woman) Male: 9 (41) Nonbinary: 1 (5)	22	Unspecified (clinical decision support tools)	Priority applications of AI in primary care Impact of AI on primary care provider roles Considerations for provider training in AI

a AI: artificial intelligence.

b PCP: primary care physician.

### Quality of Included Studies

Based on the quality assessment, all included studies were considered high quality, as they met most of the CASP criteria, with 1 study [47] fulfilling all the criteria. Many of the included studies [1,2,12,17,46] provided limited or no information regarding the CASP 6 item.

### Main Findings

The thematic synthesis resulted in 3 main themes (Textbox 1): “The Relationship with and Actions of AI Systems,” “Implementing AI Responsibly,” and “Training Physicians and Artificial Minds.”

Textbox 1. **Analytical themes and descriptive themes.**

**The Relationship With and Actions of Artificial Intelligence (AI) Systems**

- Accessibility
- Nonverbal communication
- The changing patient-physician relationship
- AI and clinician collaboration

**Implementing AI Responsibly**

- Data safety
- Data sharing
- Implementation of AI
- Regulations for AI

**Training Physicians and Artificial Minds**

- Bias
- Diversity in data
- Inaccurate data
- Reinforcing prejudice

#### Theme 1: The Relationship With and Actions of AI Systems

The included studies highlight patients’ attitudes toward the changing patient-physician relationship, nonverbal communication, AI and clinician collaboration, accessibility, AI accuracy, efficiency, and priority applications of AI in primary care.

Many participants asserted the importance of an emotional connection with their physician [1,47], noting that body language helped them feel supported [2,47] and trust their physicians’ advice more [47]. Concerns were raised about AI’s assumed inability to perceive emotions, potentially affecting care quality when relying solely on nonemotional health data [2,47], highlighting physicians’ unique ability to identify underlying issues that patients might not verbally express [47]. Some patients expressed that AI could enhance emotional comfort during times of difficult health challenges and contribute to acceptance [12].

Accessibility was a common concern [1,2,12,46,47]. Many patients considered AI to be beneficial in rural areas [12]. Patients expressed concerns regarding the accessibility of AI in health care, particularly for individuals with unique characteristics, such as those who use sign language, have strong accents, or have atypical sensory processing [12]. Some thought AI could increase health care costs [46], and overdiagnosis was mentioned as a reason [1].

Patients expressed trust in the health system and their physicians [1,12,47]. In 1 study [47], all participants said they would follow their physician’s screening recommendations. Primary care physicians were considered authoritative figures [1], and many patients trusted them more than AI [2,47], citing a general distrust of technology and the belief that a trustful relationship isn’t possible with a machine [47]. Patients believed physicians aimed to avoid mistakes that could harm their professional reputation [47]. However, concerns have been raised that health care providers might become overly dependent on AI [2,46], potentially replacing physicians and weakening the patient-physician relationship [1]. Another concern was that primary care physicians could shift from being health care providers to mere data collectors if AI gained too much influence [1].

Opinions on the accuracy of AI systems varied. Some noted that AI’s access to vast medical data could enhance accuracy, stating that machines are more accurate than physicians [12,47], while others questioned its ability to understand nuanced symptoms [12]. Many patients doubted that AI could replace physicians, especially in decision-making, believing the patient-physician relationship is inherently human and essential to patient-centered care [2]. While some admitted AI’s superior accuracy, they still preferred human involvement in treatment [47] and viewed AI as a helpful support tool [2,47].

Patients also discussed AI’s capacity to manage large datasets and hoped it could use existing information effectively. They emphasized the importance of having as much data as possible when making decisions [12]. Suggested key AI applications in primary care included clinical documentation, practice operations, risk identification, and triage, with the potential to reduce administrative tasks and enhance physician efficiency [2].

#### Theme 2: Implementing AI Responsibly

Patients expressed opinions on regulations for AI, implementation of AI, data sharing, and data safety.

Participants called for AI regulations to prevent potential harm. Many patients viewed physicians as responsible for their care and preferred that physicians ensure the safety of AI. Patients wanted their physician to make treatment decisions and oversee ongoing care [46]. Patients believed they should have the right to decide if AI tools are used in their care [1] but also to correct their recommendations if they believed them to be inaccurate [46]. Patients were uncomfortable relying entirely on AI-generated recommendations without the ability to independently assess the reasoning behind those suggestions [17,46]. While some opposed AI acting autonomously, others were open to it, provided their physician verified its recommendations [2,46].

An urge for carefulness was described when implementing AI in health care. According to patients, the AI tools should be well tested and accurate before being used [46]. Patients acknowledged that AI would not be acceptable to all [17], but that acceptance of new technology comes gradually [1,12].

Patients’ views on health care and non-AI health technology were shaped by their social context, including identities, communities, and experiences with past illnesses. They supported AI if it aligned with medicine’s goal of curing illnesses [12] and if the design, implementation, and use of AI should preserve or strengthen the patient-physician relationship [2].

Ethical concerns were raised, such as AI’s ability to detect future illnesses, which could increase anxiety and hinder living in the present [1]. Other concerns involved the influence of business interests on the development of AI. One patient noted that health care systems have commercial interests, and companies seek to profit from their data [12]. Opinions on technological advancements were mixed, with some feeling it was beyond their control and a few expressing discomfort with technology, avoiding cell phones or the internet as long as possible [12].

Concerns about data safety included technological failures, hacking risks, and the possibility of data being reidentified after anonymization [46]. Patients identified factors that would make them more comfortable sharing data, including having unremarkable medical records, assurance of anonymization, and trusting their primary care physician as an authority. The purpose of data sharing was also important [1].

In one study [1], all patients expressed willingness to share data for AI implementation and the use of AI in general practice. However, sensitive data, such as mental illness or early retirement information, made them more skeptical about data sharing. Patients feared data misuse by insurance companies, potentially leading to monitoring or loss of liberty [8]. They desired transparency about what data were collected and wanted access to the data used for risk assessment [17].

#### Theme 3: Training Physicians and Artificial Minds

Perspectives on various aspects of training AI models, including bias, data diversity, risks of inaccurate data reinforcing prejudice, and the training of physicians in using AI, emerged among the included studies.

Patients expressed concerns about the risk of developers incorporating their biases into the datasets used to train AI [46]. Concerns were also raised that training AI with data that lacks diversity could harm certain social groups [12]. An example provided was when medical records contain errors, thereby creating a risk that AI might use incorrect information [46].

Participants acknowledged that implementing AI in health care could impact different populations unevenly, potentially reinforcing stigma or restricting access. Some shared instances where they or someone they knew faced discrimination due to mental illness, alcoholism, weight, or profession. They expressed ideas about AI exacerbating or alleviating these stigmas [12].

A fear was described, entailing that future generations of physicians might lack essential skills if AI applications or training fail to preserve the core competencies necessary for patient safety and patient-centered care. One patient wondered how AI would be monitored, how physicians would be trained, and how we would ensure that patients still benefit from experience and knowledge, rather than just dependency on AI [2].

## Discussion

### Principal Findings

This review presents newly emerged concepts derived from a synthesis of evidence across several studies, all using different approaches but set in a primary care context. The theme “The Relationship with and Actions of AI Systems” explored changes in the physician-patient relationship, nonverbal communication, AI-physician collaboration, accessibility, AI accuracy, efficiency, and key AI applications in primary care. Through the analysis, it became evident that patients are concerned about the prospect that AI might change the dynamics of the physician-patient relationship and that trusting AI is different from the trust placed in physicians. The part of trust placed in physicians is based on the emotional connection between patient and physician [1,2,47], but also on the knowledge that physicians have something to lose (ie, their professional reputation [47]. It is not obvious how this sense of trust nor the delegation of responsibility for one’s health could be transferred from physician to AI systems, which is why this should be carefully considered by AI developers and health care organizations implementing AI into clinician workflows. Moreover, many patients expressed feeling supported by seeing their physicians’ body language [2,47], something that may be hard to replicate using AI. Patients shared their views on AI regulations, implementation, data sharing, and data safety under the theme “Implementing AI Responsibly.” This theme illustrates the varied views patients hold concerning the implementation of AI. Some patients reported that they considered it necessary for health care providers to inform them about the use of AI [1], which is not routinely done in current health care. For many years, physicians have received computationally automated decision support on a daily basis, such as when ordering an electrocardiogram for the analysis of heart-related conditions [52]. Consequently, physicians and health care providers should be aware of the discrepancy between current practice and patients’ expectations and consider that patients’ preferences may differ depending on their social context, identity, and lived experience [12]. The last theme, “Training Physicians and Artificial Minds,” studies highlighted perspectives on training AI models, focusing on issues such as bias, data diversity, the risk of inaccurate data reinforcing prejudice, and the training of clinicians in AI use. A knowledge gap was identified, as none of the included studies specifically addressed opinions on LLMs.

### Comparison With Prior Work

Patients expressed trust in the current health care systems and valued their relationships with their physicians. Trust in physicians stemmed from their professional reputation and experience, qualities that patients felt a machine could not provide. Earlier studies have shown great trust in primary care physicians, aligning with our findings [53-58].

While AI as a diagnostic and decision support tool was well-accepted by patients, they strongly preferred that physicians be involved in the process. A similar sentiment was observed in a study representative of the Dutch population, where 78% of women did not fully support independent use of AI-based diagnostics in screening mammography without the involvement of a radiologist [59]. Another study found that patients preferred physicians over AI for all clinical tasks except for treatment planning based on current scientific evidence. When there was a disagreement between physicians and AI regarding diagnosis and treatment planning, most patients favored the physician’s judgment. AI was considered more acceptable when supervised by a physician, especially in diagnosis, than when used independently [60].

Patients emphasized the importance of establishing an emotional bond with their physician, which made them feel recognized and supported, thus building trust and easing decisions about whether to trust their physician. However, concerns were raised about AI’s inability to recognize emotions and how this limitation could impact care, especially when it depends solely on nonemotional health data. Concerns about the lack of human touch in AI have been mentioned in previous studies [61,62]. On the contrary, a study comparing physician and AI chatbot responses showed that chatbot responses were rated as more empathetic and of better quality than physician responses [63]. In this systematic review, we found that some participants viewed health care AI as a source of relief or hope in cases where a clear diagnosis has been elusive or where standard treatments have failed, a seemingly unique finding.

Patients were concerned that using flawed data for training AI systems could harm certain social groups and lead to unequal care. Evidence of racial bias has indeed been observed in previous studies. One such study found that, despite being assigned the same risk levels by an algorithm, Black patients were actually sicker than their White counterparts, a bias resulting in over half of Black participants being overlooked for additional care [64].

Our findings complement previous work, such as the scoping review by Moy et al [65], which broadly mapped patients’ perspectives on AI across various health care settings and study designs. While their review provides a valuable overview, our meta-synthesis offers a more focused contribution by synthesizing qualitative interview studies conducted specifically in primary care. This narrower scope enabled a deeper exploration of context-specific themes and patient experiences within primary care. Furthermore, our synthesis identified a notable gap, where none of the included studies addressed LLMs, highlighting an area for future research as these technologies become increasingly integrated into primary care workflows.

### Strengths

To our knowledge, this is the first qualitative meta-synthesis that synthesizes primary care patient perspectives on AI.

To ensure the study’s quality, two authors independently handled the selection, quality assessment, and coding. Thematic analysis was conducted in such a way as to identify if nonpatient perspectives influenced the results.

During the inclusion and exclusion process, discussions emerged regarding the definitions of AI and how it was explained to patients in different studies. The varying definitions of AI presented challenges, as the authors’ interpretations influenced the selection of studies and the summaries of the types of AI used. To maintain an inclusive approach, we aimed to include studies focusing on all types of AI. In some cases, studies addressed AI in health care more broadly. In these instances, inclusion was based on whether the recruitment and thematic framing were anchored in primary care. For example, studies that recruited patients following a recent primary care visit and analyzed their perspectives in that context were considered eligible. This approach ensured relevance to primary care while acknowledging the interdisciplinary nature of AI applications in health care.

### Limitations

A major limitation of this review is that none of the included studies explicitly addressed primary care patients’ views on LLMs, highlighting a gap in the current qualitative research on this highly relevant subject. Another limitation of this study is that the initial search included only 2 databases, PubMed and Scopus. These were selected based on the assumption that they would cover relevant literature within the study’s scope. To ensure that no eligible studies were overlooked, we later conducted an expanded search on July 10, 2025, including Web of Science, CINAHL, and PsycINFO covering publications from inception up to February 5, 2024. The extended search did not identify any additional relevant studies, which supports the comprehensiveness of the original search strategy. The initial PRISMA flowchart of study selection is listed in Multimedia Appendix 5.

The definition of primary care varies globally. In Scandinavia, it primarily refers to appointments with general practitioners, while in the United States, it encompasses all non-emergency care. The results might have differed if more studies from general practice clinics were included. However, it is reasonable to assume that patients in general practice clinics share similar views about AI as those in primary care settings, as these clinics also provide care akin to primary care.

The inclusion of teleophthalmology as part of primary care may raise questions about scope. However, in the Pelayo et al [47] study, the service was embedded in a primary care setting and used to support chronic disease management. We therefore considered the patients’ perspectives on this AI-supported referral process to be relevant to primary care broadly.

A subgroup analysis of the results was not possible due to missing information about sexes and ages linked to specific quotations. Only 1 study by Upshaw et al [2] provided age-related information alongside participant quotes. The number of studies was insufficient to support comparisons by country.

Two of the studies conducted by Richardson et al [12,46] appeared to have overlapping study participants, which is a potential source of bias. However, when extracting patient quotes and comparing the material, the quotations in the 2 papers were not identical, and the studies focused on different research questions. In qualitative research synthesis, all quotations are considered valuable and important. Repetition of similar statements does not make a particular quote more important than another. Therefore, the risk of bias in this case is expected to be low.

Many of the included studies [1,2,12,17,46] provided limited or no information regarding CASP 6 item “Has the relationship between researcher and participants been adequately considered?” In contrast to quantitative research, where methodological details, such as double-blinding are commonly reported, qualitative studies less frequently describe the relationship between the interviewer and participants. Our interpretation is that this reflects a general reporting tendency rather than a lack of quality. A “Can’t tell” rating does not imply that a study is of poor quality but rather that the relevant information was vague.

### Future Directions

Understanding patients’ perspectives is crucial as AI becomes more integrated into primary care. Future research could investigate how patients interpret various AI concepts and compare their opinions on AI across different health care settings, such as general practice clinics and primary care environments. Conducting studies in diverse countries and cultures is necessary to capture a broad range of patient attitudes toward AI in primary care, acknowledging that cultural and systemic differences may influence perceptions.

Patients have expressed concerns that machines can make mistakes and lack the authority and reputation that physicians possess. These opinions may be consciously or unconsciously influenced by a preference for social status. This raises an intriguing question: Could AI eventually achieve a form of social status and earn professional credibility? This is an important factor in determining patients’ perspectives regarding responsibility and accountability if autonomous AI commits medical malpractice. Currently, no studies have investigated this topic, highlighting a valuable avenue for future research. Finally, a notable gap remains in the literature, as few studies [27-30] have explored patient perspectives on AI in general practice settings, and none have specifically addressed LLMs.

### Conclusions

Primary care patients recognize that AI can enhance efficiency in primary health care by assisting physicians with administrative tasks and offering decision support through access to comprehensive and accurate medical data. Patients value the human elements in their relationship with physicians, considering these essential for patient-centered care. Although some acknowledge AI’s superior accuracy, they still prefer human involvement in treatment. Many patients trust their physician more than AI and doubt that AI can replace physicians. Patients advocate for physicians to ensure AI safety and promote robust frameworks for its implementation. They emphasize the importance of preserving patient choice and autonomy. Understanding patients’ perspectives and needs is vital for designing effective and user-friendly AI solutions. Understanding the risks associated with AI is essential to ensure its safe and ethical implementation in health care settings.

## Supplementary material

10.2196/72211 Multimedia Appendix 1 Artificial intelligence definitions.

10.2196/72211 Multimedia Appendix 2 Search strings.

10.2196/72211 Multimedia Appendix 3 Codebooks.

10.2196/72211 Multimedia Appendix 4 Sociodemographic and study characteristics.

10.2196/72211 Multimedia Appendix 5 Initial PRISMA (Preferred Reporting Items for Systematic Reviews and Meta-Analyses) flowchart of study selection.

10.2196/72211 Checklist 1 PRISMA checklist.

10.2196/72211 Checklist 2 CASP checklist.

## Abbreviations

AI: artificial intelligence
CASP: Critical Appraisal Skills Programme
CDSS: clinical decision support system
DL: deep learning
LLM: large language model
MeSH: Medical Subject Headings
ML: machine learning
NLP: natural language processing
OECD: Organization for Economic Co-operation and Development
PRISMA: Preferred Reporting Items for Systematic Reviews and Meta-Analyses
PROSPERO: International Prospective Register for Systematic Reviews
SPIDER: sample, phenomenon of interest, design, evaluation, research
WHO: World Health Organization
