# Supplementary material for: Exploring Primary Care Patients’ Perspectives on Artificial Intelligence: Systematic Literature Review and Qualitative Meta-Synthesis
Source: JMIR AI. 2025 Nov 19;4:e72211. doi: 10.2196/72211 (PMC12629519; doi:10.2196/72211)
Supplement: Multimedia Appendix 1 [file ai-v4-e72211-s001.doc]

**Multimedia Appendix 1**

AI definitions

At the most foundational level of abstraction, there is a division between machine learning (ML) based models and more traditional rules-based AI, often called Good Old Fashion AI (GOFAI)[1]. One of the first AI-driven healthcare applications, MYCIN, was based on a rules-based system, and this type of technology continues to coexist alongside the more innovative but complex ML-based systems [2]. To the left of the figure, a series of circles delineate ML-based technologies. ML uses algorithms to learn patterns, and by training on large data sets it can make predictions based on the training data. DL, a complex form of ML using multiple layers of artificial neuronscan predict outcomes based on many different variables [3]. Generative AI (GAI), is a type of AI using deep-learning techniques. GAI can generate new content including text, video, music, images and speech [4]. The most granular form of AI defined here is represented by LLMs, which is one instantiation of GAI[5]. LLMs involve the use of NLP and DL to interpret and generate human language based on the input they receive [4]. To the right of the figure, GOFAI is depicted. As mentioned, the MYCIN algorithm was a type of GOFAI, but so are many commonly used calculators often incorporated into electronic health records (EHRs), such as those for body-mass index or scripts controlling for interactions between different medications [2]. Natural Language processing (NLP), and clinical decision support systems (CDSS), are overarching concepts that could be based on either ML or GOFAI architectures [6]. NLP recognizes and analyzes text and speech [3], and allows computers to interpret and generate human language [7].


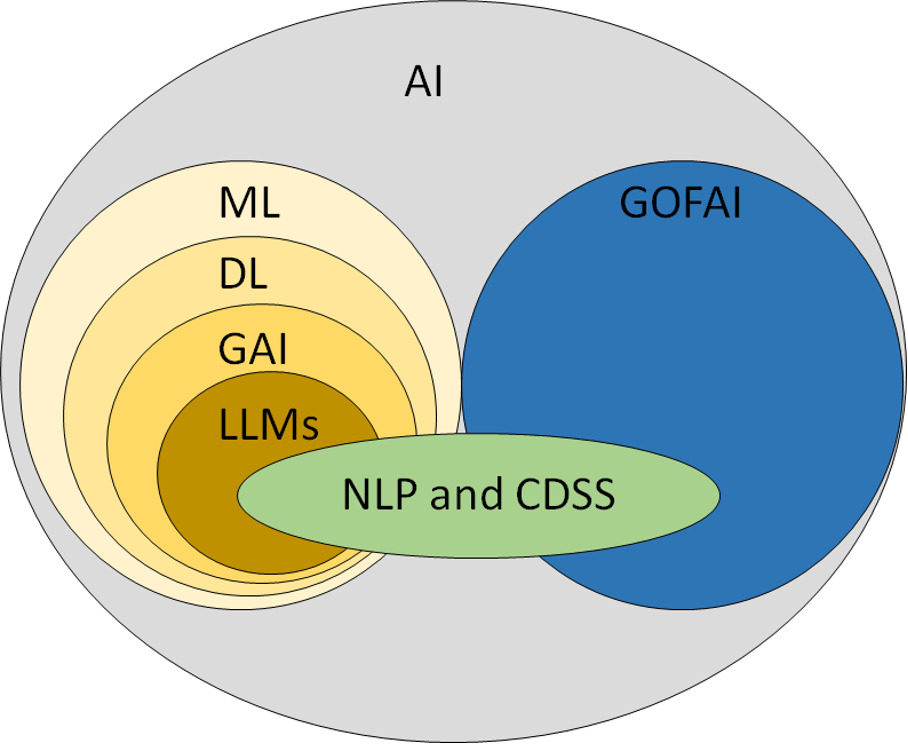


***Figure 1: Various AI systems with overlapping characteristics***

***AI- Artificial Intelligence, ML- Machine Learning, DL- Deep Learning, GAI- Generative Artificial Intelligence, LLMs- Large Language Models, GOFAI- Good Old Fashion AI, NLP- Natural Language Processing, CDSS- Clinical Decision Support Systems***

1. Tedre M, Toivonen T, Kahila J, Vartiainen H, Valtonen T, Jormanainen I, et al. Teaching Machine Learning in K–12 Classroom: Pedagogical and Technological Trajectories for Artificial Intelligence Education. IEEE Access. 2021;9:110558-72. doi: 10.1109/ACCESS.2021.3097962.

2. Shortliffe EH. Computer-based medical consultations: MYCIN. Journal of Clinical Engineering 1976;1:69-88. doi: 10.1097/00004669-197610000-00011.

3. Davenport T, Kalakota R. The potential for artificial intelligence in healthcare. Future Healthc J. 2019 Jun;6(2):94-8. PMID: 31363513. doi: 10.7861/futurehosp.6-2-94.

4. Yu P, Xu H, Hu X, Deng C. Leveraging Generative AI and Large Language Models: A Comprehensive Roadmap for Healthcare Integration. Healthcare. 2023;11(20):2776. doi: 10.3390/healthcare11202776.

5. Barreto F, Moharkar L, Shirodkar M, Sarode V, Gonsalves S, Johns A, editors. Generative Artificial Intelligence: Opportunities and Challenges of Large Language Models. Intelligent Computing and Networking; 2023 2023//; Singapore: Springer Nature Singapore.

6. Wasylewicz ATM, Scheepers-Hoeks AMJW. Fundamentals of Clinical Data Science: Springer open; 2019.

7. Milne-Ives M, de Cock C, Lim E, Shehadeh MH, de Pennington N, Mole G, et al. The Effectiveness of Artificial Intelligence Conversational Agents in Health Care: Systematic Review. J Med Internet Res. 2020 Oct 22;22(10):e20346. PMID: 33090118. doi: 10.2196/20346.
