# Supplementary material for: Exploring Primary Care Patients’ Perspectives on Artificial Intelligence: Systematic Literature Review and Qualitative Meta-Synthesis
Source: JMIR AI. 2025 Nov 19;4:e72211. doi: 10.2196/72211 (PMC12629519; doi:10.2196/72211)
Supplement: Multimedia Appendix 2 [file ai-v4-e72211-s002.doc]

**Multimedia Appendix 2**

Search strings for the primary search  ***PubMed search string***

("Primary Health Care"[Mesh] OR "General Practice"[Mesh] OR "family practi*" OR"family medicine") AND ("Artificial Intelligence"[Mesh] OR "chatGPT" OR "GPT" OR"deep learning" OR "neural network" OR "machine learning" OR "natural language processing" OR "large language model*" OR "generative AI") AND (experienc* OR perception* OR impression* OR opinion* OR attitud* OR reaction* OR perspectiv* OR satisfactio* OR sentimen* OR standpoin* OR reflectio* OR conceptio* OR expectatio*)

Results: 620

***Scopus search string***

TITLE-ABS-KEY ("primary health care" OR "primary care*" OR "general practi*" OR"family practi*" OR "family medicine") AND TITLE-ABS-KEY ("artificial intelligence*"OR "AI" OR "chatGPT" OR "GPT" OR "deep learning" OR "neural network" OR "machine learning*" OR "natural language processing*" OR "large language model*" OR "generative AI") AND TITLE-ABS-KEY (experienc* OR perception* OR impression* OR opinion* OR attitud* OR reaction* OR perspectiv* OR satisfactio* OR sentimen* OR standpoin* OR reflectio* OR conceptio* OR expectatio*)

Results: 340

Date of search: February 5, 2024
Total hits (Primary Search): 960

-----------------------------------------------------------------------------------------------------------------

Search strings for the additional search

***CINAHL search string***

(( (MH "Primary Health Care+") OR (MH "Family Practice+") OR (MH "General Practice+") OR TI ("family practi*" OR "family medicine" OR "primary care") OR AB ("family practi*" OR "family medicine" OR "primary care") ) AND ( (MH "Artificial Intelligence+") OR (MH "Machine Learning+") OR (MH "Natural Language Processing+") OR TI ("chatGPT" OR "GPT" OR "deep learning" OR "neural network*" OR "machine learning" OR "natural language processing" OR "large language model*" OR "generative AI") OR AB ("chatGPT" OR "GPT" OR "deep learning" OR "neural network*" OR "machine learning" OR "natural language processing" OR "large language model*" OR "generative AI") ) AND ( TI (experienc* OR perception* OR impression* OR opinion* OR attitud* OR reaction* OR perspectiv* OR satisfactio* OR sentimen* OR standpoin* OR reflectio* OR conceptio* OR expectatio*) OR AB (experienc* OR perception* OR impression* OR opinion* OR attitud* OR reaction* OR perspectiv* OR satisfactio* OR sentimen* OR standpoin* OR reflectio* OR conceptio* OR expectatio* OR view*) )

Results: 84

***PsycINFO search string***

(DE "Primary Health Care" OR DE "General Practice" OR "family practi*" OR "family medicine") AND (DE "Artificial Intelligence" OR "chatGPT" OR "GPT" OR "deep learning" OR "neural network" OR "machine learning" OR "natural language processing" OR "large language model*" OR "generative AI") AND (experienc* OR perception* OR impression* OR opinion* OR attitud* OR reaction* OR perspectiv* OR satisfactio* OR sentimen* OR standpoin* OR reflectio* OR conceptio* OR expectatio*)

Results: 17

***Web of Science search string***

TS=(("primary health care" OR "primary healthcare" OR "primary care" OR

"family practice" OR "general practice" OR "family practi*" OR "family medicine")

AND

("artificial intelligence" OR "chatgpt" OR "gpt" OR "deep learning" OR

"neural network*" OR "machine learning" OR "natural language processing" OR

"large language model*" OR "generative ai")

AND

(experienc* OR perception* OR impression* OR opinion* OR attitud* OR reaction* OR

perspectiv* OR satisfactio* OR sentimen* OR standpoin* OR reflectio* OR

conceptio* OR expectatio* OR view*))

Results: 345

Date of search: July 10, 2025

Filters applied: Publications dated on or before February 5, 2024, across all databases.

Total hits (additional search): 446

-----------------------------------------------------------------------------------------------------------------

Total studies included in the review (primary search and additional search): 1406
