# Supplementary material for: Exploring Primary Care Patients’ Perspectives on Artificial Intelligence: Systematic Literature Review and Qualitative Meta-Synthesis
Source: JMIR AI. 2025 Nov 19;4:e72211. doi: 10.2196/72211 (PMC12629519; doi:10.2196/72211)
Supplement: Multimedia Appendix 3 [file ai-v4-e72211-s003.doc]

**Multimedia Appendix 3**
Codebooks exported from NVIVO

Primary care patients’ perspectives of AI in primary care

| Themes, Subthemes and Codes | References in NVivo | |
| --- | --- | --- |
| **Training Physicians and Artificial Minds** |  | |
| **Bias** |  | |
| AI might reinforce existing biases in health care dataset | 1 | |
| **Diversity in data** |  | |
| Concerns that data that trains AI tools may not be appropriately diverse | 1 | |
| Harmfulness to specific race and gender | 3 | |
| **Inaccurate data** |  | |
| AI becomes more accurate the more it is being used | 1 | |
| Concerns about development of health care AI using flawed datasets | 1 | |
| Fear that AI will be trained with inaccurate health data | 1 | |
| Training AI with incorrect data | 1 | |
| **Reinforcing prejudices** |  | |
| Concerns about the absorption of prejudices and effect on medical evaluation | 2 | |
| New forms of discrimination | 1 | |
| Overweight patients said providers ignore doing tests due to overweight and there is a fear AI would take after | 1 | |
| Potentially amplify or nullify stigmas | 1 | |
| **The Relationship with and Actions of a Robot** |  | |
| **Accessibility** |  | |
| Concerns about accessibility to patients with communication challenges | 1 | |
| Concerns about capabilities of including atypical individuals | 1 | |
| Increasing healthcare costs and insurance coverage | 5 | |
| Opinions differed on AI and accessibility in rural areas | 1 | |
| Overdiagnosis leads to unnecessary expenses | 1 | |
| Patients don't mind paying | 2 | |
| Removes important barriers to Access | 1 | |
| Telehealth in remote areas | 1 | |
| Will people with intellectual disability be excluded | 1 | |
| **Nonverbal communication** |  | |
| Comfort in AI |  | |
| AI could give hope or relief where conventional therapies have failed, or a definitive diagnosis is missing | 1 | |
| Computers are perfect | 1 | |
| Helpful when it comes to acceptance | 1 | |
| Multiple doctor visits | 1 | |
| Creativity |  | |
| Concerns about thinking outside the box | 2 | |
| Could be used in many different ways | 1 | |
| Importance of body language |  | |
| Importance of emotional connection | 3 | |
| Inability to read body language and read emotions | 3 | |
| **The Changing Physician-Patient Relationship** |  | |
| Distrust in AI |  | |
| A patient with chronic pain cannot distinguish between her various pains and believes that AI cannot do so either | 1 | |
| Distrust of technology | 1 | |
| Inevitable and out of their control | 1 | |
| lack of trust for AI | 1 | |
| Trustful relationships cannot be built with AI | 2 | |
| Experience with current health care influence perspectives on AI | 1 | |
| Patients view of the health care system |  | |
| Accessibility and Responsiveness |  | |
| Difficulties maintaining relationships with primary care | 1 | |
| Documentation overload | 1 | |
| Confidence in Care |  | |
| GP an authority person | 1 | |
| Greater trust in physicians | 2 | |
| Health systems are trusted | 1 | |
| Human capacity is valued | 1 | |
| Patient trust doctors more than AI because doctors have a reputation and an education | 1 | |
| Patients follow recommendations from their physician | 2 | |
| PCP examination is very important | 1 | |
| Physicians avoid making mistakes | 1 | |
| Pressure to please their GP | 1 | |
| Trust their GP | 4 | |
| You have no choice then to trust the health care system | 1 | |
| Empathy and understanding |  | |
| Physicians have empathy | 1 | |
| The role of medicine |  | |
| Evidence-based medicine is praised | 1 | |
| Scientiﬁc progress is the core of medicine | 1 | |
| The goal of medicine is to cure people of illness and maintain good health | 1 | |
| What roll does the GP play? |  | |
| Becoming overly dependent on AI | 1 | |
| Fear AI might continue this trend and further distract the attention of providers to their computers | 1 | |
| Trust in authority is decreasing | 1 | |
| Will GP's lose their roles | 2 | |
| **AI and clinicians collaboration** |  | |
| Accuracy of AI |  | |
| Ability of AI to draw connections and highly accurate predictions | 1 | |
| AI has access to all information and is therefore considered to be more accurate | 1 | |
| Cannot provide a precise diagnosis | 1 | |
| More accurate when diagnosing | 3 | |
| Teleophthalmology and eye diseases detection | 1 | |
| Uncertainty regarding the accuracy | 1 | |
| Clinical decision-support |  | |
| Choosing Human Doctors with AI Assistance for Final Diagnoses |  | |
| Preference for a human physician to review the eye photos vs. AI |  | |
| I would prefer a physician | 1 | |
| Ophthalmologist has more experience | 1 | |
| Ophthalmologists have more experience although the computer is more correct | 1 | |
| Patient feels safer when a physician is involved in the care | 1 | |
| Patients preferred physicians to be involved when reviewing their eye photos | 1 | |
| Would choose the ophthalmologist due to more experience, and the human part is involved | 1 | |
| Preference for a human physician to use AI for decision support, but make the ﬁnal diagnosis |  | |
| Most patients felt comfortable with physicians using AI as a decision support tool | 1 | |
| Detection of future illness | 4 | |
| Time Management and Efficiency |  | |
| Clinical benefits |  | |
| AI could have prevented over testing | 1 | |
| AI is thought to be time efﬁcient | 6 | |
| Immediate results | 2 | |
| Patient wishes to streamline questionnaires | 1 | |
| Potentially prevent physician and nurse burnout | 1 | |
| Tool of preventing multiple visits to specialists | 1 | |
| Contribution to over documentation | 1 | |
| Efficiency in Data Processing |  | |
| Analyzing large amount of data | 1 | |
| Hope that AI can capitalize on existing data | 1 | |
| More information leads to better decisions | 3 | |
| Virtual vs reality, navigating the decision for in person visits |  | |
| Framing expectations on AI technologies | 1 | |
| Logistical benefits of teleophthalmology | 2 | |
| Perspectives on virtual care |  | |
| Discomfort with virtual care | 1 | |
| Virtual appointments are helpful when struggling with mental illness | 2 | |
| Virtual care can make patients uncomfortable | 1 | |
| Virtual care is convenient and time efﬁcient | 1 | |
| Virtual care is inadequate | 1 | |
| Virtual care is inadequate when a patient needs a physical exam | 1 | |
| Virtual care limits the interpretation of body language | 1 | |
| Virtual care should only be used when in-person care is not possible | 2 | |
| Preference for in-person visits when they get tests done | 1 | |
| Preference for teleophthalmology over in person dilated eye exam | 1 | |
| Preferences for physical visit | 2 | |
| preferred eye exam in person | 1 | |
| Teleophthalmology complements the in-person physical exam by the PCP | 1 | |
| **Implementing AI Responsibly** |  | |
| **Data safety** |  | |
| Data recreated after anonymization | 1 | |
| Hacking into AI systems | 1 | |
| Worry about system level crash | 1 | |
| **Data sharing** |  | |
| Barriers to data sharing |  | |
| Early retirement and mental illness is considered sensitive data | 1 | |
| Fear of insurance companies' access to data | 1 | |
| Mental illness is considered sensitive data | 1 | |
| Sensitive data and impacts on data sharing | 1 | |
| Data used for deprivation of liberty | 1 | |
| Exceptions of data sharing | 1 | |
| Facilitators for data sharing |  | |
| Anonymization facilitates information sharing | 1 | |
| Data sharing is fine as long as it is not personal data | 1 | |
| Data should not be shared without consent | 1 | |
| Patients feel comfortable Sharing data with their GP | 1 | |
| Patients that viewed their GP as an authority felt safer sharing data | 1 | |
| Patients were willing to share data for the development and implementation of AI in general practice | 1 | |
| Purpose behind data sharing was relevant for the patients | 1 | |
| Sharing health data is fine | 1 | |
| **Implementation of AI** |  | |
| Acceptance comes gradually |  | |
| People are used to other technologies and with time it could be the same with AI | 1 | |
| Thinks AI will be accepted | 1 | |
| Caution in developing and implementation of AI | 2 | |
| Ethical Considerations |  | |
| Commercial interests and impacts on AI implementation | 2 | |
| Ethical dilemma | 1 | |
| Detecting future illness, an ethical dilemma | 1 | |
| Recommendations of ai and influence on insurance coverage | 2 | |
| Technological advancement | 3 | |
| AI is still evolving | 1 | |
| AI technology to perform feats they previously thought impossible | 2 | |
| Everything was better before | 2 | |
| Outsmarted by AI | 2 | |
| Technological progress is not always considered a good thing | 2 | |
|  |  | |
| Computers take over our whole civilization, references from movies | 1 | |
| Fear of AI taking over the world | 1 | |
| Patients' engagement with AI |  | |
| Compatible with the goals of medicine | 1 | |
| Everyday technology and associations with medical AI |  | |
| Connections between healthcare ai and non-medical technology | 1 | |
| Framing healthcare AI as an advanced version of online checkers | 1 | |
| Healthcare AI framed as an advanced version of familiar online tools | 1 | |
| Patients contextualized healthcare ai trough their experiences with non-health technology | 1 | |
| Smart phones are considered a form of AI | 2 | |
| Generational differences |  | |
| Generational perception of AI | 1 | |
| Older patients feel uncomfortable with ai compared to younger patients | 1 | |
| Social factors influence patients' engagement | 1 | |
| Personal experiences of illness affect perceptions of AI |  | |
| AI was framed in relation to past illness experiences | 1 | |
| Past experiences illustrate how ai would not have been helpful | 1 | |
| Prior encounters with medicine shaped engagement with hypothetical AI innovations | 1 | |
| Supporting ai depends on alignment with the values ​​of medicine | 1 | |
| **Regulations for AI** |  | |
| Assurances about safety is wanted | 1 | |
| Autonomy |  | |
| AI should not have the ability to act autonomously | 1 | |
| Our choice decides the future of AI | 1 | |
| Patients want to be able to correct AI | 1 | |
| Preservation of patient choice and autonomy | 1 | |
| Economic interests influencing regulations of AI | 1 | |
| Patients expect their clinicians to ensure AI safety |  | |
| AI tools can have more authority, but patients prefer their providers to check on AI | 1 | |
| Healthcare providers retaining final discretion over treatment and responsibility | 1 | |
| I believe the doctor always has the responsibility | 1 | |
| Patients expect their clinicians to ensure AI safety | 1 | |
| Wants a human to check on AI | 1 | |
| Regulatory protections against potential harms | 2 | |
| Isolating primary care patients’ perspectives from studies with mixed perspectives | |  |
| Themes, Subthemes and Codes | | References in Nvivo |
| **Training Physicians and Artificial Minds** | |  |
| Concerns about how doctors would be trained | | 1 |
| Concerns future provider generations could fall victim to deskilling if AI applications or AI training does not preserve core skills | | 1 |
| Concerns on how to ensure that patients get the benefits of experience and knowledge | | 1 |
| training must prepare providers to use AI safely and effectively while retaining clinical skills | | 1 |
| **The Relationship with and Actions of a Robot** | |  |
| **Accessibility** | |  |
| Concerns about limiting access for those unable to understand technology if input from stakeholders is not considered. | | 1 |
| Patients were interested in remote monitoring and self-management applications | | 1 |
| **Accuracy of AI** | |  |
| The accuracy of AI is questioned | | 1 |
| **Clinical decision-support** | |  |
| Concerns providers would rely on algorithm more than their clinical reasoning | | 1 |
| Patients were not convinced that AI could ever replace providers in the context of clinical decision making | | 1 |
| **Collaboration of AI and clinicians** | |  |
| Adolescents desire resources to help themselves | | 1 |
| AI as a tool for doctors | | 1 |
| important to ensure the clinical utility of the algorithm | | 1 |
| improvement suggestions for the algorithm | | 1 |
| Prescreening | | 1 |
| Suicide cases go unnoticed because it was not recognized | | 1 |
| Time management and Efficiency | |  |
| AI could improve the efficiency and effectiveness of doctors' work | | 1 |
| AI has the potential to decrease administration | | 1 |
| Clinical benefits of the algorithm | | 1 |
| Highest priority applications of AI in primary care | | 1 |
| Patients were open for AI to alleviate the burden of certain routine tasks | | 1 |
| The algorithm feels useful | | 1 |
| The algorithm is interesting | | 1 |
| The algorithm is neat | | 1 |
| **Nonverbal communication** | |  |
| AI might not recognize problems the way a doctor who has built a trustful relationship with patients can | | 1 |
| Patient-provider relationship is intrinsically human, enabling mechanisms of patient-centered care | | 1 |
| **Implementing AI Responsibly** | |  |
| **Data sharing** | |  |
| Desire to know the data that led to risk determination | | 1 |
| Feels intrusive | | 1 |
| Wanting to know what data they get from the phone and why they need it | | 1 |
| **Implementation of AI** | |  |
| AI would not be acceptable to all | | 1 |
| Design, implementation, and use of AI should uphold or enhance the patient-provider relationship | | 1 |
| Patients and providers shared similar priority applications for AI and concerns | | 1 |
| Shared values | | 1 |
| Privacy concerns | | 1 |
| Wants the doctor to check AI | | 1 |
