# Supplementary material for: Exploring Primary Care Patients’ Perspectives on Artificial Intelligence: Systematic Literature Review and Qualitative Meta-Synthesis
Source: JMIR AI. 2025 Nov 19;4:e72211. doi: 10.2196/72211 (PMC12629519; doi:10.2196/72211)
Supplement: Multimedia Appendix 4 [file ai-v4-e72211-s004.doc]

## Multimedia Appendix 4 Sociodemographic and study characteristics

| Study | Interview date | Participants (n) | Occupation | Explanition of AI | Patient category |
| --- | --- | --- | --- | --- | --- |
| A Framework for Examining Patient Attitudes Regarding Applications of Artificial Intelligence in Healthcare [12] | October 2019 and February 2020 | 87 | Education level above a high school equivalent (87%) | Participants were given a brief definition of AI. Each group was then presented with series of case studies illustrating the diversity of ways AI might be applied in medicine. These case studies focused on the use of AI for: image analysis, optimizing preventative health, in-patient monitoring, diagnostic support, and for engagement with patients during primary-care appointments | Adult, over 18 years who had a recent primary care visit at a large academic health center in Minnesota and Wisconsin. Same participants as in the study |
| Patient Perspectives on Data Sharing Regarding Implementing and Using Artificial Intelligence in General Practice - A Qualitative Study [1] | October 2019 and January 2022 | 10 | Different range. Short - medium - to long and some students | Three vignettes were developed in written form and the patient referred to in the vignettes was fictional. 1) The intention with AI in the general practice is that it should be a helping hand in the GPs work. 2) Annette Jensen (fictional) gets asked if her health data can be shared to develop and test AI in general practice. 3) When the GP is done, he says that he will have to do some calculations on the computer that uses AI. Annette (fictional) can also see the screen while the GP lets the computer work. Even though they referred to data-driven AI, they were not explicit about this towards the interviewees, who were only presented with the term “artificial intelligence”, since they probably would not have gained any further knowledge from the information, since they did not know much about AI in general | Danish citizens above 18 years of age and registered at a general practice clinic |
| Perspectives of Latinx Patients with Diabetes on Teleophthalmology, Artificial Intelligence-Based Image Interpretation, and Virtual Care: A Qualitative Study | Between July to November 2022 | 20 | 15% Elementary school or less, 20% middle school graduate, 5% Some high school, 25% High school graduate or GED, 15% Some college or technical school, 20% collage graduate | Components from the National Institute on Minority Health and Health Disparities (NIMHD) Research Framework, were used to create questions that addressed participants’ perspectives on teleophthalmology, AI-based image interpretation and virtual care | Adults (18 years or older) who self-identified as Latinx/Hispanic and diagnosed with type 1 or type 2 diabetes at a federally qualified health center in Madison |
| Patient Apprehensions About the Use of Artificial Intelligence in Healthcare [46] | Between October 2019 and February 2020 | 87 | N (%) Less than HS Grad 1 (1.1). Grade 12 or GED 10 (11.5). College 1–3 years 31 (35.6). College 4 years or more 23 (26.4). Graduate school 22 (25.3). Education level above a high school equivalent (87%) | Participants were given a brief definition of AI, along with examples of nonmedical applications of AI. Case studies of specific uses of AI in medicine were then presented to participants for discussion and reflection. The explanation of AI given during the focus group was specific to machine learning. Focus groups 1–6 used three case studies: an image analysis tool, a ChatBot for asking questions about a medical procedure, and a risk prediction tool that analyzed and flagged patients at risk of developing a preventable condition. Focus groups 7–15 used three different cases: a complex diagnostic tool, a well-person visit involving a ChatBot, and a tool for monitoring patients in an intensive care unit (ICU) | Adult, over 18 years who had a recent primary care visit at a large academic health center in Minnesota and Wisconsin. Same participants as in the study " A framework for examining patient attitudes regarding applications of artificial intelligence in healthcare" |
| Adolescent, Parent, and Provider Perceptions of a Predictive Algorithm to Identify Adolescent Suicide Risk in Primary Care [17] | - | 31 in total; 9 adolescents 12 parents  10 providers |  | The algorithm was described as integrating data from the EHR and smartphones to predict suicide risk | Adolescents needed to be between 12–17 years of age and a patient at a Primary Care (PC) site associated with one of the two health systems involved in this research. Eligibility also required that the parent or child had discussed the child’s depression or suicidality in PC and/or the child had been screened for depression at their PC visit |
| Priorities for Artificial Intelligence Applications in Primary Care: A Canadian Deliberative Dialogue with Patients, Providers, and Health System Leaders [2] | Between September 8th and October 15th 2020 | 22 | - | AI was described to participants as a technology that automates tasks typically requiring human intelligence, such as processing information, reasoning, learning, planning actions, and communicating in natural language. AI was presented as a general-purpose prediction technology that estimates missing information from available data | Patients who (1) spoke English, (2) were aged 18 years or older, and (3) visited a primary care provider at least once within the last year were recruited through social media and patient advisory organizations |
