# Supplementary material for: Exploring Primary Care Patients’ Perspectives on Artificial Intelligence: Systematic Literature Review and Qualitative Meta-Synthesis
Source: JMIR AI. 2025 Nov 19;4:e72211. doi: 10.2196/72211 (PMC12629519; doi:10.2196/72211)
Supplement: Multimedia Appendix 5 [file ai-v4-e72211-s005.doc]

**Multimedia Appendix 5**

*
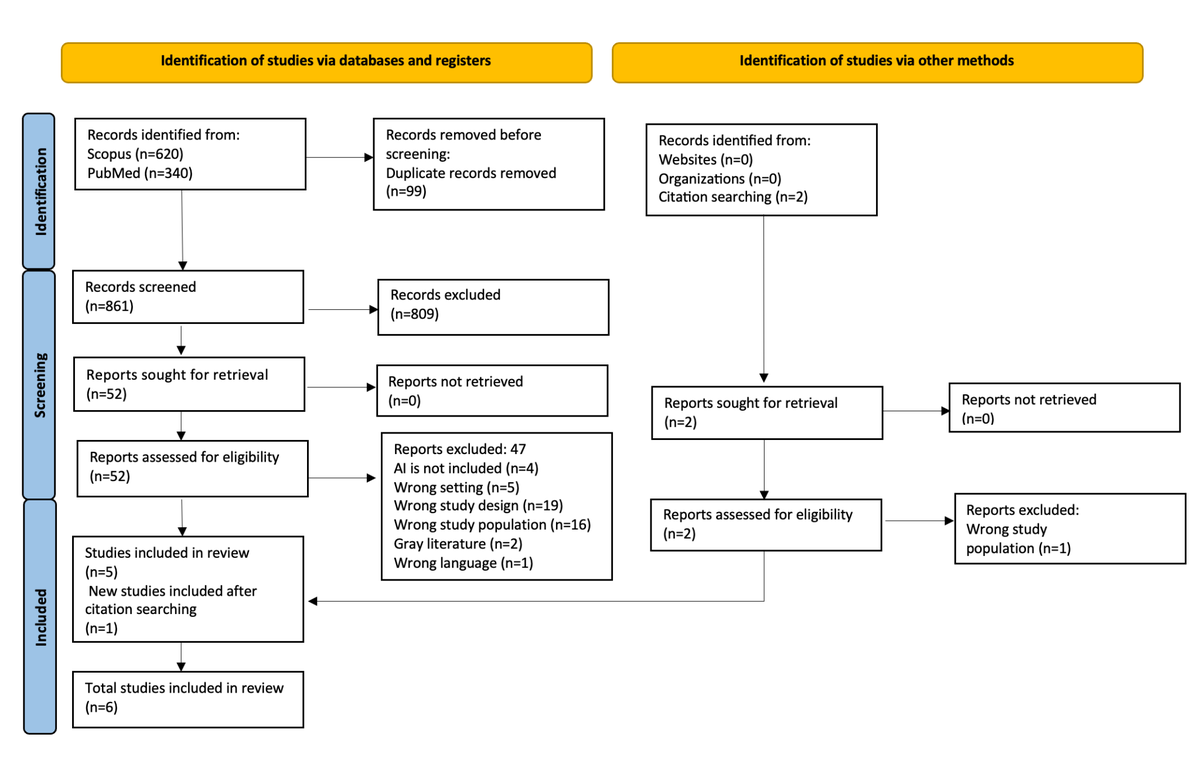
*

*Figure: Initial PRISMA (Preferred Reporting Items for Systematic Reviews and Meta-Analyses) flowchart of study selection*.
