# Supplementary material for: Exploring Primary Care Patients’ Perspectives on Artificial Intelligence: Systematic Literature Review and Qualitative Meta-Synthesis
Source: JMIR AI. 2025 Nov 19;4:e72211. doi: 10.2196/72211 (PMC12629519; doi:10.2196/72211)
Supplement: Checklist 2 [file ai-v4-e72211-s007.doc]

## Checklist 2 Critical Appraisal Skills Programme Qualitative Research Checklist, (CASP)

| Study | | CASP1 | CASP2 | CASP3 | CASP4 | CASP5 | CASP6 | CASP7 | CASP8 | CASP9 | CASP10 |
| --- | --- | --- | --- | --- | --- | --- | --- | --- | --- | --- | --- |
| A Framework for Examining Patient Attitudes Regarding Applications of Artificial Intelligence in Healthcare [12] | | YES | YES | YES | YES | YES | NO | Can't tell | YES | YES | YES |
| Adolescent, Parent, and Provider Perceptions of a Predictive Algorithm to Identify Adolescent Suicide Risk in Primary Care [17] | | YES | YES | YES | YES | YES | NO | YES | YES | YES | YES |
| Patient Perspectives on Data Sharing Regarding Implementing and Using Artificial Intelligence in General Practice - A Qualitative Study [1] | | YES | YES | YES | YES | YES | Can't tell | YES | YES | YES | YES |
| Perspectives of Latinx Patients with Diabetes on Teleophthalmology, Artificial Intelligence-Based Image Interpretation, and Virtual Care: A Qualitative Study [47] | | YES | YES | YES | YES | YES | YES | YES | YES | YES | YES |
| Priorities for Artificial Intelligence Applications in Primary Care: A Canadian Deliberative Dialogue with Patients, Providers, and Health System Leaders [2] | | YES | YES | YES | YES | YES | NO | YES | YES | YES | YES |
| Patient Apprehensions About the Use of Artificial Intelligence in Healthcare | | YES | YES | YES | Can't tell | YES | NO | YES | YES | YES | YES |
| CASP1 | Was there a clear statement of the aims of the research? | | | | | | | | | | |
| CASP2 | Is a qualitative methodology appropriate? | | | | | | | | | | |
| CASP3 | Was the research design appropriate to address the aims of the research? | | | | | | | | | | |
| CASP4 | Was the recruitment strategy appropriate to the aims of the research? | | | | | | | | | | |
| CASP5 | Was the data collected in a way that addressed the research issue? | | | | | | | | | | |
| CASP6 | Has the relationship between researcher and participants been adequately considered? | | | | | | | | | | |
| CASP7 | Have ethical issues been taken into consideration? | | | | | | | | | | |
| CASP8 | Was the data analysis sufficiently rigorous? | | | | | | | | | | |
| CASP9 | Is there a clear statement of findings? | | | | | | | | | | |
| CASP10 | How valuable is the research? | | | | | | | | | | |
